# Contribution of domain structure to the function of the yeast DEDD family exoribonuclease and RNase T functional homolog, Rex1

**DOI:** 10.1261/rna.078939.121

**Published:** 2022-04

**Authors:** Peter W. Daniels, Taib Hama Soor, Quentin Levicky, Ewald H. Hettema, Phil Mitchell

**Affiliations:** Department of Molecular Biology and Biotechnology, The University of Sheffield, S10 2TN Sheffield, United Kingdom

**Keywords:** RNA processing, nuclease, 5S rRNA, yeast

## Abstract

The 3′ exonucleolytic processing of stable RNAs is conserved throughout biology. Yeast strains lacking the exoribonuclease Rex1 are defective in the 3′ processing of stable RNAs, including 5S rRNA and tRNA. The equivalent RNA processing steps in *Escherichia coli* are carried out by RNase T. Rex1 is larger than RNase T, the catalytic DEDD domain being embedded within uncharacterized amino- and carboxy-terminal regions. Here we report that both amino- and carboxy-terminal regions of Rex1 are essential for its function, as shown by genetic analyses and 5S rRNA profiling. Full-length Rex1, but not mutants lacking amino- or carboxy-terminal regions, accurately processed a 3′ extended 5S rRNA substrate. Crosslinking analyses showed that both amino- and carboxy-terminal regions of Rex1 directly contact RNA in vivo. Sequence homology searches identified YFE9 in *Schizosaccharomyces pombe* and SDN5 in *Arabidopsis thaliana* as closely related proteins to Rex1. In addition to the DEDD domain, these proteins share a domain, referred to as the RYS (Rex1, YFE9 and SDN5) domain, that includes elements of both the amino- and caroxy-terminal flanking regions. We also characterize a nuclear localization signal in the amino-terminal region of Rex1. These studies reveal a novel dual domain structure at the core of Rex1-related ribonucleases, wherein the catalytic DEDD domain and the RYS domain are aligned such that they both contact the bound substrate. The domain organization of Rex1 is distinct from that of other previously characterized DEDD family nucleases and expands the known repertoire of structures for this fundamental family of RNA processing enzymes.

## INTRODUCTION

Stable RNAs are processed from initial transcripts by the action of one or more ribonucleases, which either cleave the phosphodiester backbone endonucleolytically or shorten the RNA exonucleolytically from the 5′- or 3′ end. The 3′–5′ exonucleolytic trimming of extended transcripts to mature stable RNAs is universally observed throughout biology. The genomes of most organisms encode a number of 3′–5′ exoribonucleases and, in the cellular systems that have been well characterized, these have been shown to have overlapping, redundant substrate specificities, and cellular functions (Deutscher 2006; Houseley and Tollervey 2009).

Eight distinct 3′ exoribonucleases have been characterized in *Escherichia coli* (Zuo and Deutscher 2001; Bechhofer and Deutscher 2019). Despite the strong functional overlap of these enzymes, there is a unique requirement for one enzyme, RNase T, in the final 3′ end trimming of some stable RNAs. Mutants lacking RNase T accumulate a major form of 5S rRNA that is extended at its 3′ end by 2 nt (Li and Deutscher 1995), while 3′ extended forms of 23S rRNA are observed that are predominantly one or 3 nt longer than the species observed in wild-type cells (Li et al. 1999). The 3′ ends of 5S and 23S rRNA share a common structural feature, where base-pairing between the 5′ and 3′ ends of the RNA generate a double-stranded region with short 3′ extensions of one or two U residues, respectively. The requirement for RNase T in the final maturation of 5S and 23S rRNA reflects its unique ability to remove nucleotides close to regions of double-stranded RNA (Zuo and Deutscher 2002c). Notably, RNase T was initially identified as the enzyme necessary for the removal of the 3′ terminal adenosine residue of deacylated tRNA (Deutscher et al. 1985) that also has a terminal stem with a short 3′ extension.

RNase T is a member of the DEDD family of 3′ exonucleases (Zuo and Deutscher 2001). The catalytic domains of these enzymes contain four conserved acidic residues (hence the term, DEDD domain) within three exonuclease motifs that coordinate binding of two Mg^2+^ cations at the active site. Mutational analyses of ribonucleases within this family have shown that each of these four residues is required for catalytic activity (Zuo and Deutscher 2002a; Phillips and Butler 2003). The DEDD family also includes the 3′ exonuclease proof-reading domains of DNA polymerase I and the ε subunit of DNA polymerase III, and the catalytic domain of DNA exonuclease I (Moser et al. 1997). It is not clear what features of the catalytic domain enable DEDD family enzymes to act on RNA or DNA but several, including RNase T, can degrade both.

Members of the DEDD family of exonucleases have distinct structural features that extend the substrate binding path of the catalytic domain. RNase T is a homodimeric protein, each subunit containing a cluster of conserved basic residues within its DEDD domain that forms a nucleotide binding site (NBS). The NBS of one monomer is positioned adjacent to the catalytic site of the other monomer, providing an extended binding site for the 3′ end of the RNA (Zuo and Deutscher 2002b; Zuo et al. 2007). In contrast, RNase D is a monomer that contains two α-helical HRDC domains in addition to the DEDD catalytic domain. The DEDD and HRDC domains of RNase D are arranged in a funnel structure that has been proposed to contribute to substrate specificity (Zuo et al. 2005).

The genome of the budding yeast *Saccharomyces cerevisiae* encodes multiple DEDD family exoribonucleases that have known functions in RNA processing and/or degradation. The best characterized of these proteins is Rrp6, a catalytic subunit of the nuclear exosome RNase complex (Allmang et al. 1999; Zinder and Lima 2017). The amino-terminal PMC2NT domain of Rrp6 forms a stable heterodimeric complex with the small basic protein Rrp47 (Stead et al. 2007; Schuch et al. 2014) and genetic depletion of either Rrp6 or Rrp47 causes a decreased level of expression of the other protein (Feigenbutz et al. 2013; Stuparevic et al. 2013). A DEDD domain is also observed in members of the yeast RNA exoribonuclease (Rex) family of proteins that includes Rex1. Rex1 (also known as Rna82 or Rnh70) is a nuclear protein (Frank et al. 1999) required for the final 3′ end maturation of 5S rRNA and is implicated in the maturation of the 3′ end of 25S rRNA, the 3′ processing of tRNAs and the renewal of the CCA terminal sequence of tRNA (Piper et al. 1983; Kempers-Veenstra et al. 1986; van Hoof et al. 2000; Copela et al. 2008; Ozanick et al. 2009). Yeast *rex1* mutants accumulate forms of 5S rRNA that are extended at their 3′ end by 2 or 3 nt (Piper et al. 1983; van Hoof et al. 2000). Rex1 therefore carries out a similar set of RNA processing reactions to those mediated by RNase T in *E. coli*. However, the two proteins are structurally distinct; the DEDD domain of yeast Rex1 is flanked by extended amino- and carboxy-terminal regions and lacks the residues that comprise the hydrophobic interaction surface or the NBS of RNase T. None of the genes encoding a DEDD family exoribonuclease in yeast is essential for cell growth. However, null alleles of the *REX1* gene are synthetic lethal with deletions of the genes encoding Rrp6 or its associated protein Rrp47 (Peng et al. 2003; van Hoof et al. 2000).

To identify features of Rex1 that are required for its function in vivo, we generated a set of *rex1* mutants and analyzed the effect on cell growth in *rrp47Δ* mutants. The *rex1* mutants were also screened for their impact on protein expression and localization, 5S rRNA processing, Rex1 RNA binding activity in vivo and exonuclease activity in vitro. These analyses reveal a requirement for both the amino- and carboxy-terminal flanking regions of Rex1 for stable expression of the DEDD domain and for substrate binding, and characterize a bipartite nuclear localization signal close to the amino terminus of the protein.

## RESULTS AND DISCUSSION

### Rex1 function is dependent upon its amino- and carboxy-terminal regions, as well as specific features in the DEDD domain

**FIGURE 1.**
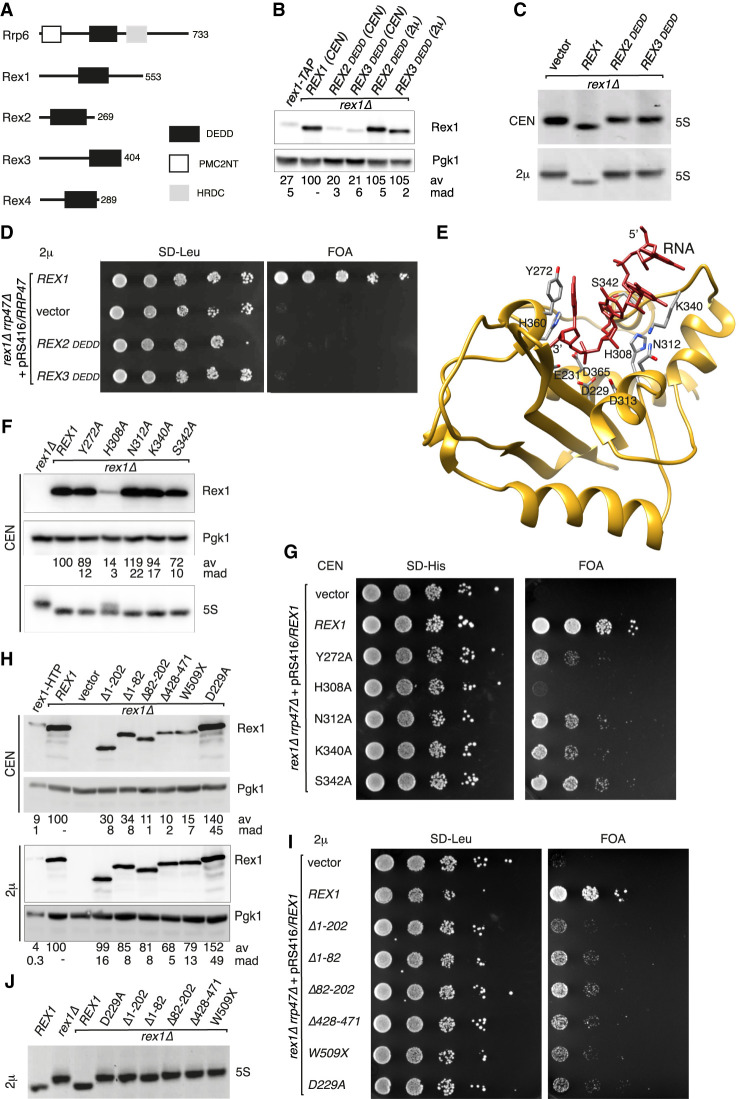
Rex1 function requires both the DEDD domain and flanking regions. (*A*) Domain organization of the yeast Rex enzymes and Rrp6. Protein lengths (amino acid residues) are indicated. (*B*) Western analysis of Rex1 fusion proteins. Rex1 was expressed either from the integrated *rex1-TAP* allele or from plasmids encoding the wild-type protein or domain swap mutants containing the DEDD domain from either Rex2 or Rex3. (*C*) Ethidium-stained RNA gels showing the relative mobility of 5S rRNA species from a *rex1Δ* mutant expressing either wild-type Rex1 or the domain swap mutants. (*D*) Plasmid shuffle assay comparing wild-type and *rex1* domain swap mutants. (*E*) Threaded model of the DEDD domain of Rex1 (residues 227–372) with bound oligoribonucleotide, derived from the Pan2/RNA structure (PDB 6R9M). Side chains of residues implicated in catalysis or substrate binding are shown. (*F*) Western analysis and 5S rRNA northern analysis of wild-type and *rex1* point mutants. (*G*) Plasmid shuffle assay of the *rex1* point mutants. (*H*) Western analysis of wild-type and *rex1* deletion mutants. (*I*) Plasmid shuffle assay of the *rex1* deletion mutants. (*J*) Northern analysis of 5S rRNA from the *rex1* deletion mutants. Expression from low copy number, centromeric (CEN) or high copy number 2 micron (2µ) plasmids is indicated in each panel. Expression levels of mutant Rex1 proteins, normalized to Pgk1 and expressed as a percentage of the wild-type protein, are indicated *beneath* each lane. Values given are the mean average (av) and the mean absolute deviation (mad) of three independent replicates.

The four Rex proteins from *S. cerevisiae* (Rex1, Rex2, Rex3, and Rex4) vary in size from 31–63 kDa, each containing a DEDD domain of approximately 160 amino acids as the only protein domain annotated in the *Saccharomyces* Genome Database (https://www.yeastgenome.org [accessed, 7-19-21]) (Fig. 1A). Rex1 is the largest of these proteins, its catalytic domain being flanked by comparably sized amino- and carboxy-terminal regions. The unique ability of Rex1 among the yeast Rex proteins to complement for the lack of Rrp6 (van Hoof et al. 2000) may in principle be due to functions associated with the amino- and/or carboxy-terminal regions of the protein or due to specific features of its catalytic domain. To address this, we first generated domain swap mutants that expressed Rex1 variants where the DEDD domain had been substituted for that from either the distantly related Rex2 or the very closely related Rex3 protein (Gerstberger et al. 2017). To assess the effect of mutations on Rex1 expression levels, wild-type and mutant Rex1 proteins were expressed as amino-terminal fusion proteins that harbor two copies of the z domain of protein A from *Staphylococcus aureus* (denoted zz). We also generated yeast strains expressing *rex1-TAP* or *rex1-HTP* (His-TEV-Protein A) alleles from the chromosomal *REX1* locus that bear the same duplicated z domain epitope (Puig et al. 1998; Granneman et al. 2009). The *rex1-TAP* and *rex1-HTP* alleles supported growth of an *rrp6Δ* mutant and did not exhibit the 5S rRNA processing phenotype characteristic of *rex1Δ* mutants, providing indicators of Rex1 expression levels at or above a functional activity threshold.

The observed levels of the chimeric Rex1 fusion proteins were lower than the wild-type Rex1 protein when expressed from centromeric plasmids in a *rex1Δ* strain but comparable to the Rex1 expression levels from a related *rex1-TAP* strain (Fig. 1B). Expression of the domain swap mutants in the *rex1Δ* strain failed to complement the 5S rRNA processing phenotype (Fig. 1C). To address whether this was due to the lower expression levels of the mutant proteins relative to the wild-type protein, we also expressed the wild-type and domain swap proteins from a high copy number, 2 micron plasmid. Using this vector, steady state expression levels of the chimeric domain swap proteins were about fourfold higher than the Rex1-TAP protein and close to that of the plasmid-encoded wild-type protein (Fig. 1B). However, the mutants still failed to complement the 5S rRNA processing phenotype (Fig. 1C). Furthermore, while expression of the wild-type *REX1* control supported growth of the *rex1Δ rrp47Δ* double mutant, transformants expressing either chimeric protein from the high copy number plasmid failed to grow (Fig. 1D). These data suggest that specific structural features of the Rex1 DEDD domain are required for the overall structural folding or stability of the protein and/or may contribute to substrate binding.

Threading of the yeast Rex1 DEDD domain sequence (residues 227–382) to the available structure of Pan2 in association with bound oligonucleotide (Tang et al. 2019) provided an excellent fit, with a RMSD for the Cα atoms of 0.75 Å. Residues involved in RNA binding in the Pan2 structure could therefore be easily mapped within the threaded Rex1 model (Fig. 1E). The aromatic ring of Y975 in yeast Pan2 stacks onto the base of the terminal 3′ nt bound in the active site of the enzyme. A tyrosine residue is also found at the equivalent position in Rex1 from *S. cerevisiae* (Y272) and in the functionally homologous Rexo5 from *D. melanogaster*, while a conservative substitution to phenylalanine is seen in human Rexo5, yeast Rex3, and RNase T. Pan2 makes several contacts with the phosphodiester backbone of the bound substrate. Side chain interactions are mediated by N1019 and S1048 of Pan2 (Tang et al. 2019), both residues of which are also found at the equivalent positions in yeast Rex1 (N312 and S342, respectively). Y1046 in Pan2 makes main chain interactions with the ribose phosphate backbone of the bound substrate, with the side chain being directed away from the RNA. The side chain of the equivalent residue in Rex1, K340, is directed toward the bound substrate in the threaded model.

To address a potential requirement for these residues in Rex1 function, alanine substitutions were generated and the mutants were assayed for complementation of the *rex1Δ rrp47Δ* growth phenotype and the 5S rRNA processing defect of the *rex1Δ* mutant. Substitution of residues Y272, N312, K340, and S342 had a minor effect on the expression of Rex1 and the corresponding mutants exhibited no 5S rRNA processing phenotype (Fig. 1F). However, these substitutions supported growth of the *rex1Δ rrp47Δ* double mutant much less effectively than the wild-type protein (Fig. 1G). Given the contacts between equivalent residues in Pan2 and bound RNA, the slow growth rate of the Y272A, N312A, and S342A *rex1 rrp47Δ* double mutants most probably reflects defects in substrate binding. We anticipate that these residues act synergistically in substrate binding, and that loss of individual contacts is not sufficient to block substrate binding completely.

We also generated an alanine substitution of H308 within the Exo II motif, the equivalent residue in mouse PARN (H280) and SDN1 from *A. thaliana* (H236) being implicated in substrate interaction (Wu et al. 2009; Chen et al. 2018). The H308A mutation caused a strong reduction in Rex1 expression levels (Fig. 1F) and a correspondingly strong impact on growth in the plasmid shuffle assay (Fig. 1G). Nevertheless, 5S rRNA processing was only partially affected in this mutant (Fig. 1F). These two observations are not inconsistent; the synthetic lethality of the *rex1Δ rrp47Δ* mutant is not due to defective 5S rRNA processing, as the block in 5S rRNA processing observed in the *rex1Δ* mutant is not exacerbated in a conditional *rex1Δ rrp47* double mutant (Garland et al. 2013). One possibility is that 5S rRNA is more readily processed by Rex1 than some of its other substrates. Alternatively, partial rather than complete processing of a Rex1 substrate other than 5S rRNA may be growth limiting in the absence of Rrp47.

To address whether regions of Rex1 other than the catalytic domain are required for its function, we generated a series of amino- and carboxy-terminal deletion mutants. We constructed three amino-terminal deletion mutants; a large deletion that essentially lacks all the polypeptide sequence upstream of the catalytic domain (Δ1–202) and two smaller deletions thereof (Δ1–82 and Δ82–202). Deletion of the carboxy-terminal region of Rex1 was predicted to cause loss of function, since allele linkage and sequence analysis of the loss of function *rna82-1* allele (Piper et al. 1983) revealed it to be congenic with *REX1* and to have a premature termination codon (W433X) downstream from the catalytic domain (van Hoof et al. 2000). We generated two carboxy-terminal *rex1* mutants; one is a truncation that lacks the final 45 amino acid residues (W509X) and the other is a deletion downstream from the catalytic domain (Δ428–471). We also mutated one of the four conserved acidic residues within the DEDD domain to an alanine (D229A). This mutation was predicted to block catalytic activity, based on previous studies on the equivalent mutation in RNase T, PARN, and Rrp6 (Ren et al. 2002; Zuo and Deutscher 2002a; Phillips and Butler 2003) and the analysis of the D229A, E231A double mutant in Rex1 (Ozanick et al. 2009).

The steady state expression levels of the Δ82–202, Δ428–471, and W509X *rex1* mutants were markedly lower than the wild-type protein when expressed from a centromeric plasmid, yet nonetheless comparable to that of the integrated *rex1* allele (Fig. 1H). To ensure that observed effects were not due to limiting expression, the mutants were also expressed from a high copy number vector. Expression levels of all mutants from this vector were comparable to that of the wild-type protein and notably higher than expression from the *rex1-HTP* allele (Fig. 1H, lower panel). Strikingly, all the *rex1* deletion mutants gave rise to only very small colonies in the plasmid shuffle assay and failed to complement the 5S rRNA processing defect of the *rex1Δ* mutant when expressed from either the centromeric plasmid or the high copy number plasmid (Fig. 1I,J). Since only very low expression levels of Rex1 are sufficient to facilitate maturation of at least some 5S rRNA in vivo (Fig. 1F), we infer that all the *rex1* deletion mutants are severely compromised in their ability to process 5S rRNA. Taken together, the data show that the structural folding and/or function of Rex1 is dependent upon features of both the amino-terminal and carboxy-terminal regions of the protein, as well as specific characteristics of its catalytic domain. As predicted from previous studies, the D229A *rex1* mutant failed to complement the 5S rRNA processing phenotype of the *rex1Δ* single mutant (Fig. 1J).

### Residues 17–52 of Rex1 constitute an NLS

**FIGURE 2.**
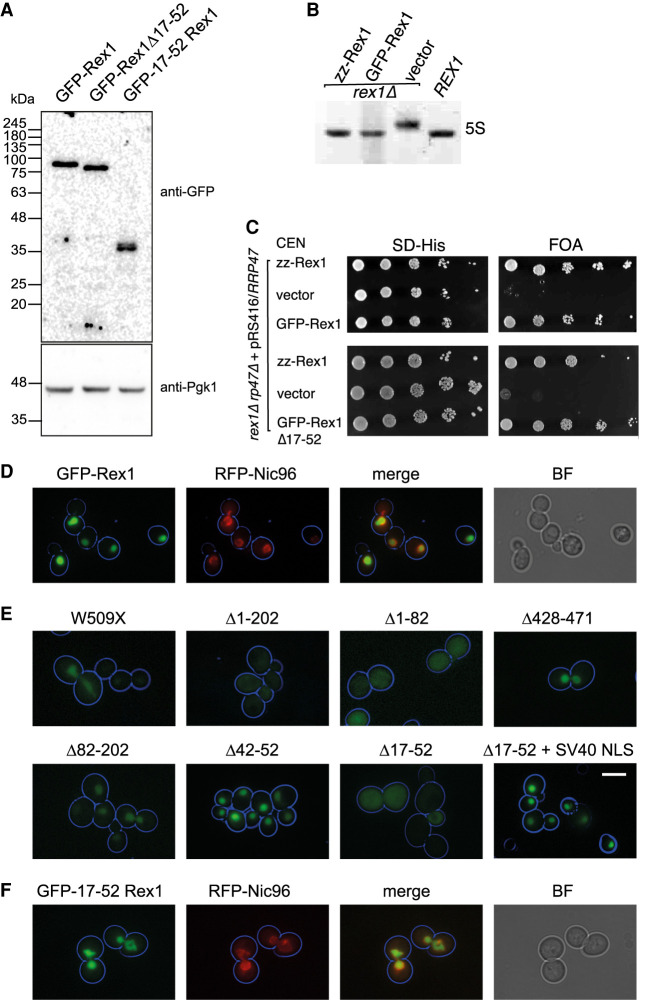
Identification of a nuclear localization signal in Rex1. (*A*) Western analysis of whole-cell extracts from a *rex1Δ* mutant expressing GFP–Rex1 fusion proteins. (*B*) Ethidium-stained RNA gel showing the relative migration of 5S rRNA species from a *REX1* wild-type strain and *rex1Δ* mutants expressing either Rex1 zz or GFP fusions. (*C*) Plasmid shuffle assays on *rex1Δ rrp47Δ* double mutants expressing Rex1 GFP and zz fusion proteins. (*D*–*F*) Epifluorescence microscopy images of cells expressing versions of GFP–Rex1 and mRFP–Nic96. (*D*) Colocalization of GFP–Rex1 and mRFP–Nic96. (*E*) Localization of GFP–Rex1 deletion mutants. (*F*) Colocalization of GFP fused to residues 17–52 of Rex1 and mRFP–Nic96. Cell circumferences are indicated in blue. Bar, 5 µm. (BF) Brightfield, (SV40-NLS) simian vacuolating virus 40 nuclear localization signal.

To determine whether the deletion mutants affect the nuclear localization of Rex1, we generated plasmid constructs encoding wild-type and mutant GFP-tagged Rex1 fusion proteins and compared their subcellular distribution by fluorescence microscopy. Western analysis showed that the wild-type GFP–Rex1 protein was stably expressed (Fig. 2A) and its expression in a *rex1Δ* mutant complemented the 5S rRNA processing phenotype (Fig. 2B). Furthermore, this construct genetically complemented the growth phenotype of the *rex1Δ rrp47Δ* double mutant (Fig. 2C). Consistent with the nuclear localization of Rex1 (Frank et al. 1999), GFP–Rex1 localized to a region coincident with the distribution of the nucleoporin RFP–Nic96 (Fig. 2D; Grandi et al. 1993). The carboxy-terminal Δ428–471 and W509X mutants also showed a localized subcellular distribution, while the amino-terminal Δ1–202 deletion gave a diffuse, nonlocalized signal (Fig. 2E). Further analysis of the amino-terminal mutants revealed that the Δ1–82 mutant showed a nonlocalized distribution throughout the cell, while the distribution of the Δ82–202 mutant was restricted in a similar manner to the wild-type protein. This data suggests that the first 82 residues of Rex1 contain a signal that mediates the nuclear localization of Rex1.

NLS prediction webserver tools indicated a potential lysine-rich NLS spanning residues 42–50 and a longer, overlapping bipartite signal between residues 17–51 (Kosugi et al. 2009; Nguyen Ba et al. 2009). We generated GFP fusion *rex1* deletion mutants that lacked residues 42–52 or 17–52 and analyzed their subcellular distribution. The Δ42-52 mutant showed a normal nuclear distribution, while the larger Δ17-52 mutant showed a nonlocalized signal throughout the cell. Addition of the NLS from the large T antigen of SV40 virus to the Δ17–52 mutant restored nuclear localization of the protein (Fig. 2E). Furthermore, addition of residues 17–52 of Rex1 to the carboxyl terminus of GFP was sufficient to direct localization of the fusion protein to the nucleus, as shown by its colocalization with Nic96 (Fig. 2F). Western analyses of GFP–Rex1, the Δ17–52 deletion mutant and the GFP–NLS fusion protein demonstrated that the localized proteins were expressed as full-length, intact polypeptides (Fig. 2A). These data demonstrate that residues 17–52 of Rex1 constitute an NLS. The amino-terminal region of the mouse and human proteins has previously been shown to harbor an NLS (Silva et al. 2017).

Notably, the Δ17–52 mutant supported growth of the *rex1Δ rrp47Δ* double mutant strain (Fig. 2C). These data suggest that, in the absence of the identified NLS, sufficient Rex1 can be localized to the nucleus through one or more additional mechanisms to support cell growth in the absence of Rrp47. A similar observation was previously made for the *rrp6-15* mutant that lacks a functional NLS and causes delocalization of the Rrp6 protein but nevertheless complements the temperature-sensitive growth phenotype of an *rrp6Δ* mutant (Phillips and Butler 2003).

Rex1 is both phosphorylated and ubiquitylated in vivo. Strikingly, the sites of phosphorylation (S24, T26, S27, and T34) are clustered within the NLS, while a nearby lysine residue (K58) is a known site of ubiquitylation (Albuquerque et al. 2008; Holt et al. 2009; Swaney et al. 2013). Phosphorylation at S24 is reduced during the environmental stress response (MacGilvray et al. 2020) and Rex1 activity has been reported to be limiting for 3′ end processing of specific tRNAs during growth at high temperature and upon nutritional deprivation (Foretek et al. 2016). This suggests Rex1 activity may be regulated in part through post-translational modifications that alter its nucleocytoplasmic distribution, in a manner similar to the RNA polymerase III repressor Maf1 (Oficjalska-Pham et al. 2006; Roberts et al. 2006).

### The amino- and carboxy-terminal regions of Rex1 are required for 5S rRNA processing in vitro

To address whether the mutant Rex1 ribonucleases are compromised in their ability to accurately process RNA, Rex1 GST fusion proteins were expressed in *E. coli*, purified by affinity chromatography and incubated with 5S RNP complexes isolated from a *rex1Δ* mutant. The substrate and reaction products were then resolved through denaturing acrylamide/urea gels, together with total cellular RNA from a wild-type strain to serve as a marker for mature 5S RNA.

**FIGURE 3.**
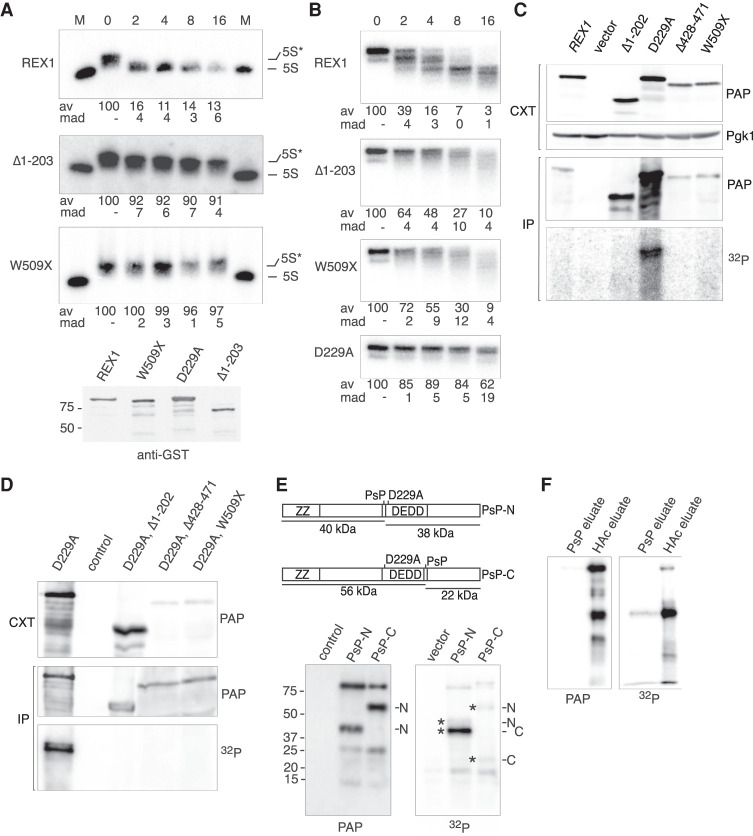
The amino- and carboxy-terminal regions of Rex1 are required for RNA binding. (*A*,*B*) In vitro nuclease assays. (*A*) 5S rRNA processing assays. (*B*) Assays using a 5′ ^32^P-labeled DNA oligonucleotide. Time points (minutes) are indicated *above* each panel. Lanes labeled M contain cellular RNA from a wild-type strain. The amount of full-length substrate (as a mean average [av] percentage of the total) remaining at each timepoint is indicated *below*, together with the mean absolute deviation (mad) values (*n* ≥ 2). The *lower* panel shows a western analysis of the purified proteins assayed. (*C*–*F*) In vivo RNA crosslinking analyses. Analyses are shown of whole-cell extracts (CXT) and purified proteins (IP). Westerns show zz-tagged proteins (labeled PAP) or the Pgk1 protein. PhosphoImaged blots are labeled ^32^P. Control samples are from a strain expressing nontagged Rex1. (*C*) Analysis of wild-type and mutant Rex1 proteins. (*D*) Analysis of deletion mutants bearing the active site mutation. (*E*) Proteolytic cleavage of Rex1/RNA complexes. PsP cleavage sites and the predicted fragments are indicated. Analyses shown are of the acid eluates after proteolysis. Asterisks denote cleavage products. (*F*) comparison of PsP eluates and acetic acid (HAc) eluates of the PsP-N construct. A fivefold relative excess of the PsP eluate over the acid eluate was analyzed.

Upon incubation with the wild-type Rex1 protein, the majority of the 3′ extended 5S rRNA was converted to a product of the same size as the mature 5S rRNA by the first time point (Fig. 3A). In contrast, very little of the substrate was shortened upon incubation with either the Δ1–203 mutant or the W509X mutant throughout the whole time course. The same observations were made upon assaying Rex1 wild-type and mutant zz fusion proteins purified from yeast extracts. These data show that Rex1 exhibits exoribonuclease activity when expressed in *E. coli* and that its ability to process a physiologically relevant substrate, 3′ extended 5S rRNA, is dependent upon both the amino- and carboxy-terminal regions of Rex1.

We also assayed the nuclease activity of recombinant Rex1 proteins using a DNA oligonucleotide substrate that is predicted to have negligible secondary structure. Wild-type Rex1 exhibited DNase activity in vitro, while the D229A mutant had only a very low activity similar to that seen for the analogous active site mutant of RNase T (Fig. 3B; Zuo and Deutscher 2002a). The Rex1 DNase activity was less processive than the 5S RNP processing activity, with discrete intermediates detected that are progressively shortened during the time course. The Δ1–203 and W509X mutants showed some catalytic activity upon incubation with DNA but the substrate was shortened by both mutants at a slower rate than that seen upon incubation with the wild-type protein.

### Rex1 makes multiple contacts with RNA

To address whether the amino- or carboxy-terminal region of Rex1 contributes to substrate binding, we compared the yield of covalent Rex1/substrate complexes generated upon UV irradiation of intact, growing cells expressing wild-type or mutant Rex1 fusion proteins. Cross-linked protein/RNA complexes were visualized by protein purification from cell lysates under stringent conditions, on-bead RNase digestion and 5′ ^32^P labeling of RNP complexes, followed by western blotting and phosphorImaging of the eluted proteins. The Rex1 mutants were expressed from multicopy plasmids to ensure expression levels comparable to that of the wild-type protein.

All fusion proteins were expressed comparably and readily recovered upon purification from lysed, irradiated cells, but crosslinking to RNA was only consistently observed for the D229A mutant (Fig. 3C). This suggests that inhibition of catalysis is required for efficient crosslinking under the experimental conditions used. We therefore introduced the D229A mutation into the Δ1–202, Δ428–471, and W509X deletion mutants and assayed the resulting double mutants, together with the D229A single mutant. Comparable amounts of each fusion protein were recovered upon purification from lysates of irradiated cells, but crosslinking to RNA was observed only for the full-length D229A mutant (Fig. 3D). We conclude from these data that both the amino- and carboxy-terminal regions of Rex1 are required, either directly or indirectly, for substrate binding in vivo.

To determine whether direct contacts are made between the amino- and carboxy-terminal regions of Rex1 and bound substrate, crosslinking experiments were performed on cells expressing amino-terminal zz fusions of Rex1 containing an internal PreScission protease (PsP) cleavage site either at the amino- or carboxy-terminal end of the DEDD domain (Fig. 3E). Crosslinked RNP complexes were treated as above and digested with PsP prior to acid elution from the beads.

Western analyses of the acid eluates revealed bands corresponding to the amino-terminal 40 and 56 kDa epitope-tagged cleavage products (labeled N in the PAP blot shown in Fig. 3E), along with residual, nondigested full-length Rex1. PhosphorImager analyses of the same blots revealed pairs of ^32^P-labeled bands corresponding to both the amino- and carboxy-terminal polypeptide fragments of each construct (denoted with asterisks and labeled N or C). The electrophoretic migration of the ^32^P-labeled PsP-N digestion products was slightly retarded in the SDS-PAGE gels, relative to the western signal from the same fragment, due to the additional mass of the crosslinked oligonucleotide. Direct comparison of the PsP-N digestion products eluted upon enzymatic cleavage with the acid eluates (Fig. 3F) confirmed that the more intensely ^32^P-labeled, faster migrating PsP-N product was the nontagged, carboxy-terminal fragment. Only a small amount of the carboxy-terminal cleavage product was solubilized upon PsP digestion. This fragment was retained on the beads after stringent washing with buffer containing 2 M MgCl_2_, which readily dissociates stable multimeric protein complexes (Allmang et al. 1999). These observations suggest that the amino-terminal flanking region of Rex1 has very stable intramolecular interactions with either the DEDD domain or carboxy-terminal flanking region, rather than comprising a structurally distinct, modular domain. Taken together, the data show that Rex1 makes direct contacts with its RNA substrates within both the amino- terminal and carboxy-terminal regions of the protein in vivo and are consistent with loss of 5S rRNA processing activity observed in the deletion mutants due to a decrease in substrate binding.

We consistently observed a higher level of ^32^P associated with the PsP-N carboxy-terminal cleavage product than would be expected, based on the observed efficiency of PsP cleavage. Preliminary studies showed that digestion using a range of buffer and incubation conditions under which PsP is active (Ullah et al. 2016) did not allow complete digestion, suggesting that a fraction of the purified PsP-N fusion protein is not amenable to cleavage. We interpret these observations to suggest that the fraction of crosslinked protein that is poorly accessible for digestion by PsP is also a poor substrate for polynucleotide kinase.

### Rex1 amino- and carboxy-terminal flanking sequences constitute a composite domain

**FIGURE 4.**
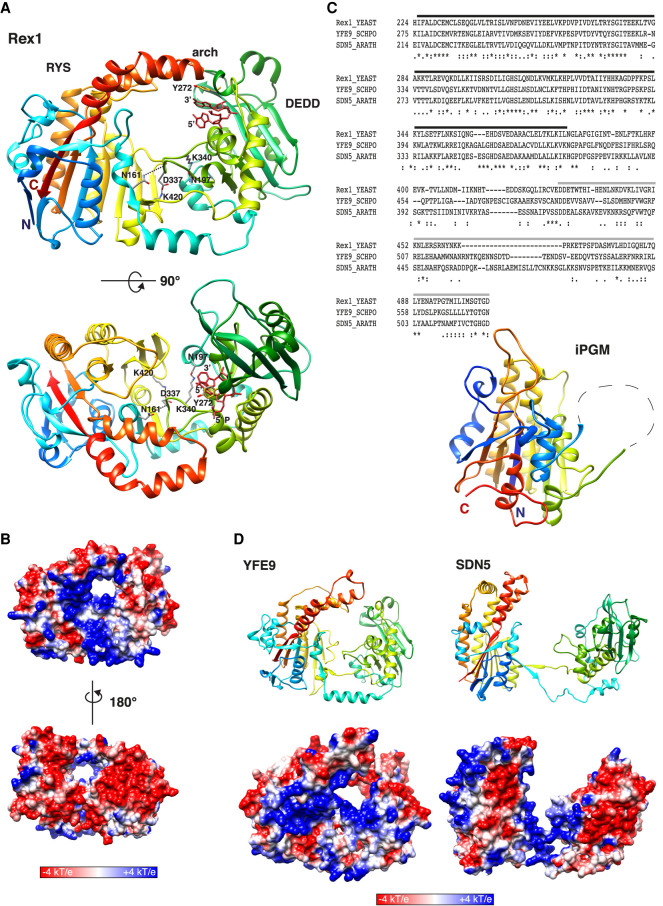
The Dual domain structure of Rex1. (*A*) Ribbon structure of the AlphaFold model of Rex1 (residues 53–553) with bound substrate. The terminal dinucleotide from the threaded Rex1/RNA model (Fig. 1E) was superposed onto the Rex1 AlphaFold model by manual alignment with residue Y272. The Y272 side chain is shown in both models (gray and gold) to indicate the alignment. Front and top views are shown. Coloring is from blue (amino terminus) to red (carboxyl terminus). The DEDD domain, the RYS domain and the helical arch are labeled. Interactions between specified residues are shown. The 3′ and 5′ nt are labeled. The 5′ phosphate group is labeled in the *lower* panel. (*B*) Electrostatic potential map of Rex1. Front and rear views are shown. (*C*) BLAST sequence alignment of Rex1, YFE9, and SDN5. The DEDD domain is overscored in black. iPGM- and PPM-related sequences are overscored in gray. The fold of the substrate binding domain of iPGM from *B. stearothermophilus* (residues 2–78 and 309–511) is shown. (*D*) Ribbon structures and electrostatic potential maps of (*left* panels) YFE9 (residues 87–631) and (*right* panels) SDN5 (residues 41–567). Amino-terminal sequences of the Rex1, YFE9, and SDN5 AlphaFold models with low confidence scores are excluded. Orientations of iPGM, YFE9, and SDN5 are shown as for Rex1 in *A*.

The AlphaFold Protein Structure Database (Jumper et al. 2021) was released during preparation of this report. The predicted structure for Rex1 consists of the catalytic DEDD domain and an adjacent domain that we refer to here as the RYS (Rex1, YFE9, and SDN5) domain (Fig. 4A). The catalytic center lies on the internal surface of the DEDD domain. A striking feature of the model is an extended α-helical arch within the RYS domain that is directed toward the DEDD domain. The model has a very high confidence score (pLDDT mean score of 91.6% for residues 53–553), with only the amino-terminal region and two loops (residues 203–209 and 462–469) that have low confidence scores. The largely unstructured amino-terminal 52 residues of Rex1 are not shown in Figure 4. The two domains are aligned by interactions involving D337, which makes both a main chain interaction with N161 and a side chain interaction with K420. The domain interface is further constrained through an interaction between K340 and N197. Whether K340 is involved in both intramolecular interactions and substrate binding is currently unclear.

Two terminal nucleotides linked by the scissile phosphate were mapped to the active site by superposition of the threaded structure of the Rex1 DEDD domain with associated substrate onto the AlphaFold model (Fig. 4A). The two models aligned well, with an RMSD of 2.0 Å for the complete length of the threaded DEDD domain. The orientation of the modeled nucleotides in the active site strongly suggests that the RNA substrate enters the active site from the front, as viewed in Figure 4. Mature 5S rRNA has a terminal stem with a single 3′ nt overhang. Molecular docking studies suggest that there is sufficient distance between the two domains in the model to accommodate base-paired nucleotides, consistent with the enzyme being able to trim the short 3′ overhang found in 5S rRNA. However, a base pair is too bulky to fit through the entrance to the active site (Fig. 4B). This strongly suggests that there is realignment of the two domains upon substrate binding. The electrostatic potential map of the model reveals an extended, positively charged surface on the front side of the protein that may function in substrate recruitment (Fig. 4B). In marked contrast, the rear side of the protein is largely negatively charged. We speculate that the two domains are positioned further apart than shown in the model when bound to the substrate such that the RNA can interact with the basic patch on the surface of the enzyme.

The eight-stranded, extended β-sheet and associated α-helices at the core of the RYS domain integrates polypeptide sequences from both the amino- and carboxy-terminal regions of Rex1 (Fig. 4A). It follows that the structural integrity of the RYS domain is predicted to be perturbed in the amino- and carboxy-terminal *rex1* deletion mutants. Two of the mutations that showed the greatest effect on Rex1 expression levels (Fig. 1H) are restricted to elements of the RYS domain; the Δ428–471 deletion spans a central β-strand and the flexible loop adjacent to the arch, while the W509X truncation removes the arch and the β-strand at the carboxyl terminus. Both mutations result in loss of Rex1 function in vivo (Fig. 1I,J), while the W509X mutation causes a loss of 5S RNP processing activity in vitro (Fig. 3). This strongly suggests that the RYS domain is critical for expression of a catalytically active DEDD domain.

### Rex1, YFE9, and SDN5 share a similar dual domain structure

BLAST searches for homologs of Rex1 revealed that YFE9 from *S. pombe* and SDN5 from *A. thaliana* show sequence homology that extends throughout the DEDD domain and substantially into the carboxy-terminal region (Fig. 4C). Phyre2 searches using Rex1, YFE9, and SDN5 aligned a homologous region within each protein (indicated with a gray bar in Fig. 4C) downstream from the DEDD domain to the three-dimensional structure of the substrate binding domain of cofactor-independent phosphoglycerate mutases (iPGMs) and phosphopentomutases (PPMs) (Jedrzejas et al. 2000; Panosian et al. 2011), two enzymes belonging to the alkaline phosphatase superfamily. iPGMs and PPMs have a similar domain organization to Rex1, consisting of a central domain and a composite domain comprising the amino- and carboxy-terminal polypeptide sequences. Strikingly, the overall fold of the composite substrate-binding domain of iPGM from *Bacillus stearothermophilus* (Fig. 4C) and PPM from *Bacillus cereus* is very similar to that predicted for the RYS domain of Rex1 but lacks the extended helical arch. Furthermore, AlphaFold models of YFE9 and SDN5 (pLDDT mean scores of 88.7% and 87.3%, respectively, for the residues shown) predict dual domain structures similar to Rex1 that include a carboxy-terminal helical arch connecting the equivalent strands within the central beta sheet of the composite domain (Fig. 4D). The YFE9 model has a positively charged surface around the predicted entrance to the active site that potentially offers an extended substrate binding surface, as in the case of yeast Rex1. There are no interactions between the DEDD and RYS domains in the AlphaFold model of SDN5 equivalent to those involving D337 in Rex1 or in YFE9, and hence the domains are not well aligned. We anticipate, however, that these domains are more closely aligned in the protein structure. Nevertheless, there is a localized surface positive charge in the SDN5 model that lines the base of the two domains. We conclude that Rex1, YFE9, and SDN5 share a common structural domain in addition to the DEDD domain, referred to here as the RYS (Rex1, YFE9, and SDN5) domain, the fold of which is related to the substrate binding domain of iPGMs and PPMs. Furthermore, the predicted structures of Rex1, YEF9, and SDN5 suggest the presence of a substrate binding site at the entrance to the catalytic center of the enzymes.

The data reported here analyze the function of a novel protein domain found within DEDD family ribonucleases. We report that the RYS domain is required for stable expression of Rex1, contributes to substrate interaction and is critical for RNA processing. The RYS and DEDD domains are predicted to cradle the binding site for the 3′ end of RNA. A conserved basic patch in Rex1-related enzymes suggests an extended interaction surface leading to the catalytic center. The dual domain structure of Rex1 and the homodimeric arrangement of RNase T therefore represent remarkably distinct structural architectures that facilitate the 3′ end processing of equivalent RNA substrates.

## MATERIALS AND METHODS

### Plasmids and strains

Yeast sequences were amplified by PCR on genomic DNA from BY4741-related strains and cloned using standard molecular biology procedures. Constructs and strains generated in this study are given in the Supplemental Data. All constructs were validated by sequencing. Centromeric and high copy number yeast expression vectors were derived from the pRS series (Sikorski and Hieter 1989; Christianson et al. 1992).

The wild-type *REX1* ORF and 3′-UTR were cloned into a construct that allows the expression of amino-terminal zz fusion proteins from the *RRP4* promoter (Mitchell et al. 1996). Site-directed mutagenesis reactions to introduce point mutations, restriction sites or cleavage sites were done using QuikChange kits (Agilent) or by PCR with divergent primer pairs using Q5 DNA polymerase (New England BioLabs). Internal deletions were created after introducing pairs of restriction sites. The *REX2* DEDD and *REX3* DEDD domain constructs were generated by homologous recombination in yeast; the zz fusion, wild-type *REX1* expression construct was linearized with *Xcm*I and cotransformed with PCR amplicons of the *REX2* and *REX3* DEDD domains that were generated using *REX1*/*REX2* and *REX1*/*REX3* splint primers. The DEDD catalytic domain of Rex1 (residues A227–L372) was replaced with that of Rex2 (V56-Q226) and Rex3 (S245-V388). DNA encoding GFP was amplified from plasmid pFA6a-GFP(S65T)-kanMX6 (Longtine et al. 1998) as an *Eco*RV-*Eco*RI fragment and substituted for the sequence encoding the zz tag after introduction of an *Eco*RV site at the second and third codon. To introduce the SV40 NLS, oligonucleotides encoding the peptide sequence IPKKKRKVD were blunt end ligated into the GFP–Rex1ΔNLS construct after digestion with *Eco*RV. The GFP–REX1–NLS construct was generated by ligating annealed, overlapping oligonucleotides encoding Rex1 residues 17–52 into the *Eco*RI site at the 3′ end of the GFP ORF. A peptide encoding the PreScission Protease site was inserted into a unique *Bgl*II site that had initially been engineered up- or downstream from the DEDD domain. The resulting constructs harbor the peptide sequence RSLEVLFQGPRS between residues H220/G221 and A366/R367 of Rex1. For expression of Rex1 proteins in *E. coli*, inserts from the yeast expression vectors were amplified and subcloned as *Bam*HI-*Eco*RI fragments into pGEX-6P1. The amino-terminal deletion construct generated (Δ1–203) expresses an in-frame fusion but lacks the *Bam*HI site. The *NIC96* ORF and 3′-UTR was amplified as an *Nsi*I-*Xho*I fragment and cloned into an mRFP expression construct (a kind gift from the Hurt laboratory, University of Heidelberg) (Ulbrich et al. 2009).

The *rex1Δ* strain was obtained from Euroscarf (University of Frankfurt). The *rex1Δ rrp47Δ* strain has been reported previously (Costello et al. 2011). The *rex1-TAP::URA3* and *rex1-HTP::URA3* strains were generated by standard homologous recombination, using amplicons derived from pBS1539 (Puig et al. 1998) or the HTP-tagging cassette (Granneman et al. 2009), respectively.

### Yeast methods

Yeast strains were routinely grown in standard rich YPD medium (2% glucose, 1% yeast extract, 2% bacto-peptone) or selective SD medium (2% glucose, 0.5% ammonium sulfate, 0.17% yeast nitrogen base and appropriate amino acids and bases but lacking either histidine or leucine) at 30°C. Transformations were carried out using standard lithium acetate protocols. Complementation analyses were performed using plasmid shuffle assays on *rex1Δ rrp47Δ* strains that are supported by a plasmid bearing either the wild-type *RRP47* gene or encoding the zz-Rex1 fusion protein. These plasmids also carry the counter-selectable *URA3* gene. Plasmid shuffle assays were performed as previously described (Feigenbutz et al. 2013). Briefly, *rex1Δ rrp47Δ* strains were transformed with a second plasmid encoding a test *rex1* allele and transformants were selected by growth on appropriate drop-out medium. Freshly grown precultures were normalized to a standard OD at 600 nm and 10-fold serial dilutions were generated in minimal selective medium. Aliquots were pinned to the surface of agar plates containing either selective SD medium or complete minimal medium containing uracil (50 µg/ml) and 1 mg/mL 5′-fluoro-orotic acid (FOA) to select for loss of the initial plasmid. Plates were incubated at 30°C for 3 to 5 d. Assays were performed on at least three biological replicates.

Western analyses were performed on cell lysates prepared under alkaline conditions as previously described (Motley et al. 2012), except that the TCA-precipitated protein was resuspended in 15 mM Tris-HCl pH 8.7, 50% urea before adding SDS-PAGE loading buffer. Blots of yeast cell extracts were probed with peroxidase/anti-peroxidase (PAP) conjugate (P1291, Sigma-Aldrich) to detect the zz fusion proteins. Anti-Pgk1 (clone 22C5D8, Life Technologies) or anti-GFP (clones 7.1 and 13.1, Sigma-Aldrich) mouse monoclonal antibodies followed by HRP-linked goat anti-mouse secondary antibody (1706516, Bio-Rad Laboratories) were used to detect the levels of Pgk1 and GFP fusion proteins, respectively. GST-tagged proteins expressed in *E. coli* were detected with a rabbit anti-GST antibody (G7781, Merck) and a goat anti-rabbit HRP conjugate (A4914, Sigma-Aldrich). Proteins were visualized by ECL using an iChemi XL GelDoc system fitted with GeneSnap software (SynGene) and quantified using ImageJ (NIH).

Total cellular RNA was prepared from yeast using a standard phenol/guanidinium hydrochloride extraction. RNA was resolved through 8% or 6% acrylamide/urea gels and visualized by staining with ethidium bromide or hybridization of northern blots with a 5S rRNA-specific probe (Schuch et al. 2014). RNA analyses were performed on at least three biological replicates.

For epifluorescence microscopy, overnight cultures were diluted to an OD_600_ of ∼0.1 and grown for 5 to 7 h in selective glucose-based medium. The cell pellet from 1mL of log phase culture was resuspended in 100 µL fresh medium and the cell suspensions were analyzed with epifluorescence, as previously described (Motley et al. 2015). Briefly, images were acquired using an Axiovert 200M (Carl Zeiss) equipped with an Exfo X-cite 120 excitation light source, band pass filters (Carl Zeiss and Chroma Technology Corp.), Plan Apochromat 63× 1.4 NA objective lens (Carl Zeiss), and a digital camera (Orca ER, Hamamatsu Photonics). Image acquisition was performed using Volocity software (PerkinElmer). Fluorescence images were collected as 0.5 mm Z stacks, merged into one plane in Openlab (PerkinElmer) and processed further in Photoshop (Adobe) by adjustment of levels. Brightfield images were collected in one plane at the center of cells, processed where necessary to highlight just the circumference of the cells and pasted into the blue channel of Photoshop.

### 5S rRNA processing assays

5S RNP complexes containing 3′ extended 5S rRNA were purified from the *rex1Δ* strain by sucrose density gradient ultracentrifugation using buffers containing EDTA (Blobel 1971; Steitz et al. 1988). Briefly, cell pellets were lysed with glass beads in a buffer comprising 50 mM HEPES pH 7.4, 50 mM KCl, 5 mM MgCl_2_, 1 mM EDTA. After clarification of the cell extract, EDTA was added to a final concentration of 3 mM and the lysate was left on ice for 15 min. The lysates were then loaded onto 10%–50% sucrose density gradients in a buffer comprising 10 mM HEPES pH 7.4, 50 mM KCl, 1 mM EDTA and centrifuged in an SW41 rotor at 36,000 rpm for 210 min. The sucrose gradients were aliquoted and MgCl_2_ added to each fraction to a final concentration of 10 mM. The fractions were then aliquoted, frozen in liquid nitrogen and stored at −80°C. The gradient fractions were screened for 5S and 5.8S rRNA by electrophoresis through acrylamide/urea gels. Fractions were used for subsequent processing assays that contained 5S rRNA but lacked detectable 5.8S rRNA.

Expression of Rex1 GST fusion proteins in *E. coli* was autoinduced using Terrific broth supplemented with 0.05% glucose and 0.2% lactose. Cultures were grown to an OD_600_ of ∼0.5 at 37°C and then transferred to 23°C and incubated overnight before harvesting. Cells were lysed in 10 mM Tris-HCl pH 7.6, 150 mM NaCl, 0.1% Tween 20 containing 100 µM PMSF, 10 µM leupeptin, and 10 µM pepstatin A, and the proteins purified by affinity chromatography using glutathione sepharose beads. After washing with lysis buffer, bound protein was eluted by incubation in lysis buffer containing 20 mM glutathione and aliquots were frozen in liquid nitrogen. The relative yields of GST fusion proteins were estimated by SDS-PAGE analysis and equivalent amounts of the full-length proteins were used in the degradation assays.

5S RNP complexes were mixed with purified GST-tagged proteins and incubated at 30°C. Reaction mixtures contained 5 mM Tris-HCl pH 7.4, 5 mM HEPES pH 7.4, 82 mM NaCl, 25 mM KCl, 0.05% Tween 20, 10 mM glutathione, 0.5 mM EDTA, 5 mM MgCl_2_, ∼10 nM 5S RNP complex, and ∼20 nM Rex1 protein. Aliquots were removed at time-points and the reaction quenched by addition of formamide gel loading buffer. The incubation mixtures were resolved through 6% acrylamide/urea gels, together with total cellular RNA from a wild-type strain, and the RNA detected by hybridization of northern blots using a 5S RNA specific probe. RNA was visualized by PhosphoImaging using a Typhoon FLA7000 (GE Healthcare) and quantified using ImageJ.

DNase assays were carried out on GST fusion proteins using a 5′[^32^P]-labeled oligonucleotide (o1165; CACGGATCCGATGAAGTGGTTGTTGTT) predicted to have negligible secondary structure (a free energy of −1.6 kcal/mol and frequency of 17% at 30°C). Assays were carried out in mixtures containing 9 mM Tris-HCl pH 7.4, 135 mM NaCl, 0.1% Tween 20, 18 mM glutathione, ∼20 nM DNA substrate and ∼30 nM Rex1 protein. Incubation mixtures recovered at different time points were resolved through 16% acrylamide/urea gels, transferred to Hybond-N^+^ membranes and the DNA was visualized by PhosphoImaging, as above. Nuclease assays using RNA and DNA substrates were performed on multiple technical repeat time courses using at least two independent samples of each purified protein.

### Crosslinking assays

Cells expressing Rex1 zz fusion proteins from high copy number plasmids were harvested in mid-log growth, resuspended in fresh medium and transferred to petri dishes to give a depth of 1–2 mm. The petri dishes were placed on an ice-cooled glass plate and irradiated with UVC light for 5 min with a pulse time of 30 sec every minute, using a CL1000 Crosslinker (UVP Plastics). Irradiated cells were harvested by centrifugation and the cell pellets stored at −80°C.

The zz fusion proteins were purified from lysates of irradiated cells by affinity chromatography using IgG sepharose beads. Cells were lysed in a buffer consisting of 50 mM HEPES pH 7.6, 150 mM NaCl, 1 mM EDTA containing 1 mM PMSF and a yeast protease inhibitor cocktail (Melford Laboratories). Clarified lysates were mixed with IgG sepharose beads for 1 h at 4°C. After washing the beads with lysis buffer, nonspecific and indirectly bound protein was eluted in lysis buffer containing 2 M MgCl_2_ (Allmang et al. 1999; Mitchell et al. 2003). The beads were then washed again with lysis buffer and incubated with buffer containing 1 µg RNase A for 30 min at 37°C. After washing with PNK buffer (50 mM HEPES pH 7.6, 10 mM MgCl_2_, 5 mM DTT), beads were incubated with 5 units of polynucleotide kinase and 3 pmol γ[^32^P]-ATP (PerkinElmer). Nonincorporated ATP was removed by washing, and protein was recovered from the beads by elution with 0.5 M acetic acid. Eluted protein fractions were concentrated by extraction with n-butanol and then precipitated in n-butanol at −80°C. After centrifugation at 15,000*g* for 30 min, protein pellets were dried and then resuspended in SDS-PAGE loading buffer containing 50% urea. Crosslinking assays were carried out on two sets of biological replicates in the wild-type and D229A background.

For PsP cleavage of the crosslinked proteins, Rex1 was purified from irradiated cells and the bound RNA was digested and radiolabeled, as described above. After removal of unbound ATP, the beads were washed in PsP cleavage buffer (50 mM HEPES pH 7.6, 150 mM NaCl) and incubated with PsP overnight at 4°C. The solubilized fraction and subsequent acetic acid eluate were then concentrated and precipitated using butanol, as described above. Samples were resolved by SDS-PAGE and transferred to Protran membranes (GE Healthcare). Radiolabeled, crosslinked proteins were detected by PhosphoImaging and the zz fusion proteins were identified by western analyses. PsP was expressed in *E. coli* (using an expression plasmid kindly provided by Stuart Wilson, University of Sheffield) and purified by glutathione sepharose affinity chromatography, as described above. PsP cleavage assays were carried out on three sets of biological replicates.

### Bioinformatics

Yeast protein sequences were obtained from the *S. cerevisiae* Genome Database (SGD) (Engel et al. 2014). Multiple sequence alignments were generated using Clustal Omega (Madeira et al. 2019). The access code for PDB files were as follows: iPGM from *B. stearothermophilus*, 1EJJ; Pan2 from *S. cerevisiae* with bound oligonucleotide, 6R9M; PPM from *B. cereus*, 3M8W. PDB files of the AlphaFold predicted protein structures were accessed at the EBI webpage (https://www.ebi.ac.uk) using Uniprot codes (Rex1, P53331; YFE9, O94443; SDN5, Q8L7M4). The DEDD domain sequence of Rex1 (SMART domain SM00479, residues 224–382) (Letunic et al. 2021) was fitted to the three-dimensional structure of yeast Pan2 with bound oligonucleotide (Tang et al. 2019) using the one-to-one threading tool of the Phyre2 web portal (Kelley et al. 2015). RMSD values between the threaded Rex1 DEDD domain model and the Pan2 substrate complex or Rex1 AlphaFold model were determined using the pairwise alignment tool hosted on the RSCB Protein Databank website (https://www.rcsb.org/alignment) by using the jFCAT (rigid) method and default parameter settings (Li et al. 2020). Molecular graphics were generated using UCSF Chimera (Pettersen et al. 2004). The terminal dinucleotide of the bound substrate in the Rex1 DEDD domain threaded model was manually superposed onto the AlphaFold model by alignment with the Y272 residue. An RNA duplex structure (PDB 1QCU) (Klosterman et al. 1999) was used for manual molecular docking of RNA base pairs to the AlphaFold model for Rex1 in UCSF Chimera. Oligonucleotide secondary structure for the DNA substrate was predicted with the RNAfold webserver (Lorenz et al. 2011), using energy parameters for DNA and a temperature of 30°C.

## SUPPLEMENTAL MATERIAL

Supplemental material is available for this article.

## Supplementary Material

Supplemental Material

## Figures and Tables

**Peter Daniels:**
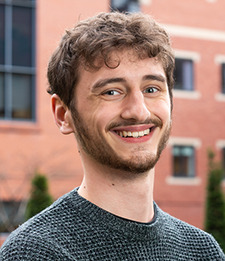


## MEET THE FIRST AUTHOR

***Meet the First Author(s)* is a new editorial feature within *RNA*, in which the first author(s) of research-based papers in each issue have the opportunity to introduce themselves and their work to readers of *RNA* and the RNA research community. Peter Daniels is co-first author of this paper, “Contribution of domain structure to the function of the yeast DEDD family exoribonuclease and RNase T functional homolog, Rex1,” along with Taib Hama Soor. Peter is a third year PhD student in Phil Mitchell's laboratory at the University of Sheffield. His research focus is the mechanistic dissection of the Rex1 exoribonuclease in baker's yeast, which fits into the laboratory's broader aim of understanding eukaryotic RNA degradation.**

**What are the major results described in your paper and how do they impact this branch of the field?**

We present mutational analyses of Rex1 that reveal previously unseen functional contributions within and beyond the annotated exonuclease domain. Most notably, we present data that supports the existence of a putative novel RNA-binding “RYS” domain, the structure of which has been predicted with high confidence in the AlphaFold Database. The striking structural features of this putative domain hint at a mechanism for substrate recognition by Rex1, and deciphering its function will represent a large step toward understanding the function of Rex1 and its homologs.

**What led you to study RNA or this aspect of RNA science?**

I find the complexity of how the cell sculpts its RNA pool fascinating, and the sheer number of uncharacterized proteins involved in the process leaves plenty of scope for new discoveries. I feel very lucky to be able to work in a field where so many pioneering discoveries have been made, yet plenty of exciting questions remain to be explored. In light of recent events it feels like there's a massive future for RNA science. We're growing closer to understanding the complexities of previously elusive disease mechanisms, and harnessing our knowledge to develop surreal new technologies.

**During the course of these experiments, were there any surprising results or particular difficulties that altered your thinking and subsequent focus?**

I'd say the biggest twist during the preparation of this paper was the release of the Rex1 structure prediction in the AlphaFold Database. By mid-July 2021 we had finished our final experiments, and one of our findings was that the entirety of the amino- and carboxy-terminal regions were essential to stable Rex1 expression—although it was hard to conceptualize the biological basis of this observation without structural data. Then, when AlphaFold DB went live on the evening of the 22nd, the structural model that they'd predicted for Rex1 explained our observations perfectly: the amino- and carboxy-terminal regions weaved into a single interdependent domain. Our truncation mutants all resulted in loss of expression because they were removing strands from the middle of an interdependent β-sheet. The excitement I felt that evening was immense—it was like all of our observations suddenly slotted into place!

**What are your subsequent near- or long-term career plans?**

If all goes to plan, I'll be aiming to submit my PhD thesis in autumn 2023. After that, I'd really like to find a post-doc position focusing on RNA metabolism, or within the broader umbrella of nucleic acid biology. My ultimate aim is to be familiar with enough techniques in the field that I'm able to study any protein in RNA biology that I come across!

**How did you decide to work together as co-first authors?**

Taib and I were both PhD students in the Mitchell laboratory, although Taib had already graduated and moved on by the time I'd started. The aim of my PhD project is to pick up where Taib left off, and this paper represents the fruits of our continuous efforts from Taib's PhD up until the third year of mine!
