# Supplementary material for: Contribution of domain structure to the function of the yeast DEDD family exoribonuclease and RNase T functional homolog, Rex1
Source: RNA. 2022 Apr;28(4):493–507. doi: 10.1261/rna.078939.121 (PMC8925975; doi:10.1261/rna.078939.121)
Supplement: Supplemental Material [file supp_28_4_493__DC1.html]

Supplemental Material 

# Contribution of domain structure to the function of the yeast DEDD family exoribonuclease and RNase T functional homolog, Rex1

## Supplemental Material

- Supplemental\_Data.docx
