# Supplementary material for: Contribution of domain structure to the function of the yeast DEDD family exoribonuclease and RNase T functional homolog, Rex1
Source: RNA. 2022 Apr;28(4):493–507. doi: 10.1261/rna.078939.121 (PMC8925975; doi:10.1261/rna.078939.121)
Supplement: Supplemental Material [file supp_078939.121_Supplemental_Data_.docx]

Supplementary Data

Strains used in this study

Key Allele Number Genotype Source

____________________________________________________________________________________________________________________

*rex1-TAP* P1837 *Mata his3∆1 leu2∆0 met15∆0 ura3∆0 rex1-TAP::URA3* This study

*rex1-HTP* P2100 *Mata his3∆1 leu2∆0 met15∆0 ura3∆0 rex1-HTP::URA3* This study

*rex1-HTP rrp6∆* P2101 *Mata his3∆1 leu2∆0 met15∆0 ura3∆0 rex1-HTP::URA3 rrp6∆::KanMX4* This study

*rrp6∆*  P781 *Mata his3∆1 leu2∆0 met15∆0 ura3∆0 rrp6∆::KanMX4*  Euroscarf

*rex1-TAP rrp6∆* P1601 *Mata his3∆1 leu2∆0 met15∆0 ura3∆0 rrp6∆::KanMX4 rex1-TAP::URA3* This study

*rex1∆*  P550 *Mata his3∆1 leu2∆1 lys2∆0 ura3∆0 rex1∆::KanMX4*  Euroscarf

*rex1∆ rrp47∆ + RRP47* P596 *Mata ade2 ade3 his3 leu2 trp1 ura3 rex1∆::KANMX4 rrp47∆::KanMX4* + p265 Costello et al., 2011

*rex1∆ rrp47∆ + REX1* P1604 *Mata his3∆1 leu2∆0 met15∆0 ura3∆0 rex1∆::KANMX4 rrp47::HphMX4* + p659 This study

Plasmids made in this study

Allele Number Vector Description

_______________________________________________________________________________________________________________

*REX1* p659 pRS416 zz-tagged wild-type Rex1.

*REX1*  p675 pRS415 zz-tagged wild-type Rex1.

*REX1*  p752 pRS425 zz-tagged wild-type Rex1.

*REX2*_DEDD_ p786 pRS313 zz-tagged Rex1 with DEDD domain from REX2.

*REX2*_DEDD_ p795 pRS425 zz-tagged Rex1 with DEDD domain from REX2.

*REX3*_DEDD_ p787 pRS313 zz-tagged Rex1 with DEDD domain from REX3.

*REX3*_DEDD_ p796 pRS425 zz-tagged Rex1 with DEDD domain from REX3.

*rex1* Y272A p920 pRS313 zz-tagged Rex1 containing the Y272A mutation.

*rex1* H308A p922 pRS313 zz-tagged Rex1 containing the H308A mutation.

*rex1* N312A p921 pRS313 zz-tagged Rex1 containing the N312A mutation.

*rex1* K340 p919 pRS313 zz-tagged Rex1 containing the K340A mutation.

*rex1* S342 p923 pRS313 zz-tagged Rex1 containing the S342A mutation.

*rex1* ∆1-202 p701 pRS415 zz-tagged Rex1 with the ∆1-202 deletion.

*rex1* ∆1-202 p719 pRS425 zz-tagged Rex1 with the ∆1-202 deletion.

*rex1* ∆1-82 p713 pRS415 zz-tagged Rex1 with the ∆1-82 deletion.

*rex1* ∆1-82 p720 pRS425 zz-tagged Rex1 with the ∆1-82 deletion.

*rex1* ∆82-202 p706 pRS415 zz-tagged Rex1 with the ∆82-202 deletion.

*rex1* ∆82-202 p748 pRS425 zz-tagged Rex1 with the ∆82-202 deletion.

*rex1* ∆428-471 p716 pRS415 zz-tagged Rex1 with the ∆82-202 deletion.

*rex1* ∆428-471 p721 pRS425 zz-tagged Rex1 with the ∆428-471 deletion.

*rex1* W509X p680 pRS415 zz-tagged Rex1 with the W509X deletion.

*rex1* W509X p705 pRS425 zz-tagged Rex1 with the W509X deletion.

*rex1* D229A p679 pRS415 zz-tagged Rex1 with the D229A mutation.

*rex1* D229A p754 pRS425 zz-tagged Rex1 with the D229A mutation.

GFP-*REX1* p775 pRS313 GFP-tagged wild-type Rex1.

RFP-*NIC96* p823 pRS416 mRFP-tagged Nic96.

GFP-*rex1* ∆1-202 p779 pRS313 GFP-tagged Rex1 with the ∆1-202 deletion.

GFP-*rex1* ∆1-82 p780 pRS313 GFP-tagged Rex1 with the ∆1-82 deletion.

GFP-*rex1* ∆82-202 p782 pRS313 GFP-tagged Rex1 with the ∆82-202 deletion.

GFP-*rex1* ∆428-471 p781 pRS313 GFP-tagged Rex1 with the ∆428-471 deletion.

GFP-*rex1* W509X p778 pRS313 GFP-tagged Rex1 with the W509X deletion.

GFP-*rex1* ∆42-52 p799 pRS313 GFP-tagged Rex1 lacking residues 42-52.

GFP-*rex1* ∆17-52 p804 pRS313 GFP-tagged Rex1 lacking residues 17-52.

GFP-*rex1* ∆17-52 + SV40 NLS p835 pRS313 GFP-tagged Rex1 lacking residues 17-5 & containing the NLS from SV40.

GFP-*rex1* 17-52 p817 pRS313 GFP fused to residues 17-52 of Rex1.

GST-Rex1 p872 pGEX6P-1 GST-tagged, wild-type Rex1.

GST-*rex1* ∆1-203 p881 pGEX6P-1 GST-tagged Rex1 with the ∆1-203 deletion.

GST-*rex1* W509X p877 pGEX6P-1 GST-tagged Rex1 with the W509X deletion.

GST-*rex1* D229A p880 pGEX6P-1 GST-tagged Rex1 with the D229A mutation.

*rex1* ∆1-202 D229A p887 pRS425 zz-tagged Rex1 with the D229A and ∆1-202 mutations.

*rex1* ∆428-471 D229A p888 pRS425 zz-tagged Rex1 with the D229A and ∆428-471 mutations.

*rex1* W509X D229A p886 pRS425 zz-tagged Rex1 with the D229A and W509X mutations.

*rex1* D229A PsP-N p934 pRS425 zz-tagged Rex1 with the D229A mutation and N-terminal PsP cleavage site.

*rex1* D229A PsP-C p935 pRS425 zz-tagged Rex1 with the D229A mutation and C-terminal PsP cleavage site.

Reference

Costello JL, Stead JA, Feigenbutz M, Jones RM and Mitchell P. 2011. The C-terminal region of the exosome-associated protein Rrp47 is specifically required for box C/D small nucleolar RNA 3’-maturation. J. Biol. Chem. **286**: 4535-4543.
